# COVID-19 exposes the urgent need for coding of outpatient neurology episodes

**DOI:** 10.1136/bmjno-2020-000080

**Published:** 2020-08-11

**Authors:** Mike Kemp, Fran Biggin, Rejith Dayanandan, Jo Knight, Hedley C A Emsley

**Affiliations:** 1 Department of Neurology, Lancashire Teaching Hospitals NHS Foundation Trust, Preston, Lancashire, UK; 2 Faculty of Health and Medicine, Lancaster Medical School, Lancaster University, Lancaster, Lancashire, UK

**Keywords:** clinical neurology

Early in the COVID-19 pandemic response, the Chief Medical Officer for England commissioned the National Health Service (NHS) Digital to identify vulnerable people at ‘high risk’ of complications from COVID-19, who should be ‘shielded’ for at least 12 weeks (shielded patient list (SPL)). The SPL was defined as a subset of circa 1.5 million patients in certain categories deemed to be ‘extremely vulnerable’ and who were advised to practice ‘shielding’, not leaving the home other than for essential healthcare needs and stopping all contact with those outside their home; these patients would need additional support from local government and health services. A larger ‘at-risk’ group (circa 19 million) normally at risk from influenza was advised to practice strict social distancing. The SPL categories included people on immunosuppression therapies sufficient to significantly increase the risk of infection, which would encompass some patients with neurological conditions (eg, multiple sclerosis), but otherwise patients with neurological diagnoses were not initially included in the SPL.

NHS Digital has acknowledged challenges in deriving the SPL, including that existing datasets did not hold data in the required form to identify the SPL, and data held in clinical codes did not directly map to the requirements in the SPL (the absence of clinical coding for many outpatient episodes does not seem to have been acknowledged). The lack of direct mapping to the SPL led to expert clinicians (via clinical specialty organisations) being asked to ‘translate’ (or map) so that individual patients could be identified. The Association of British Neurologists (ABN) produced stratification guidance at the request of organisations coordinating the identification of these patients. However, the lack of routine outpatient coding to underpin this exercise was not, seemingly, acknowledged. The general limitations of the approach, however, were apparently recognised, including the inaccuracy of the underlying centrally held administrative data, the incompleteness of the underlying data, and the speed at which the list was required (initially within 48 hours). These limitations were to be mitigated by local clinical services and general practitioners being able to add to the SPL directly.

Deficiencies in neurology informatics in the UK have been recognised for some time. The majority of clinical neurology activity takes place in the outpatient setting, but despite this, clinical coding of outpatient episodes is not mandatory. This, and the nuanced nature of identifying whether patients with particular neurological diagnoses were ‘extremely vulnerable’ based on certain specific clinical features and/or disease severity, meant that clinicians were required to manually review thousands of individual case records. Inevitably, each clinician will have approached this task somewhat differently, including the determination of ‘extremely vulnerable’, given the ABN risk stratification guidance, although detailed, did require interpretation at an individual patient level. The ABN guidance on risk stratification was, by necessity, revised repeatedly during the risk stratification exercise due to evolving information available, adding a layer of complexity. Risk stratification, although essential, represented an enormous demand (ultimately spanning several weeks) just as clinician time became even more scarce due to factors such as sickness, self-isolation and redeployment. With the benefit of coded outpatient episodes, the entire process could have been streamlined, probably with partial automation, and with a targeted approach to stratification based on diagnostic coding. The multitude of information systems, lack of consistency in file systems, names and formats, and so on further hampered efforts at the local level to automate any aspect of the process.

Pre-COVID-19, neurology outpatient coding had been recognised as a priority by the Neurology Intelligence Collaborative, a subcommittee of the National Neuroscience Advisory Group. Preliminary efforts were already underway, with the support of the Association of British Neurologists. Coding is also deemed to be complementary to the Getting It Right First Time NHS improvement programme. This exercise is now more pressing. It needs to be clinically led and driven, a crucial aspect to develop and maintain clinician ‘buy in’. There is recognition that different hospitals are at very different points in their digital maturity; the diversity of electronic patient record systems, independent or commercial, freedom of local IT teams to implement changes and so on all add to the challenge of implementation. Commitment from clinicians will be key in order to drive the process of integration of a pragmatic system of clinical classification of outpatient episodes.

Neurology services in the UK are in the midst of a ‘perfect storm’: an ageing population, burgeoning neurodegenerative disease, growing societal expectations, diminishing confidence among non-neurologists to manage neurological conditions, all conspiring to outstrip clinical neurology capacity. There was already a pressing need to implement outpatient neurology coding. COVID-19 has exposed just how urgent this issue has become. Hopefully, widespread clinical engagement will be forthcoming.

